# LRRC19—A Bridge between Selenium Adjuvant Therapy and Renal Clear Cell Carcinoma: A Study Based on Datamining

**DOI:** 10.3390/genes11040440

**Published:** 2020-04-17

**Authors:** Yitong Zhang, Jiaxing Wang, Xiqing Liu

**Affiliations:** 1 Department of Biochemistry and Molecular Biology, Harbin Medical University, Harbin 150081, China; zhangyitong@hrbmu.edu.cn; 2 The State Key Laboratory of Networking and Switching Technology, Beijing University of Posts and Telecommunications, Beijing 100876, China; jx19882008@163.com

**Keywords:** LRRC19, selenium, selenoprotein, GPX3, DIO1, renal clear cell carcinoma, KIRC, selenium adjuvant therapy, fatty acid degradation

## Abstract

Kidney renal clear cell carcinoma (KIRC) is the most common and fatal subtype of renal cancer. Antagonistic associations between selenium and cancer have been reported in previous studies. Selenium compounds, as anti-cancer agents, have been reported and approved for clinical trials. The main active form of selenium in selenoproteins is selenocysteine (Sec). The process of Sec biosynthesis and incorporation into selenoproteins plays a significant role in biological processes, including anti-carcinogenesis. However, a comprehensive selenoprotein mRNA analysis in KIRC remains absent. In the present study, we examined all 25 selenoproteins and identified key selenoproteins, glutathione peroxidase 3 (GPX3) and type 1 iodothyronine deiodinase (DIO1), with the associated prognostic biomarker leucine-rich repeat containing 19 (LRRC19) in clear cell renal cell carcinoma cases from The Cancer Genome Atlas (TCGA) database. We performed validations for the key gene expression levels by two individual clear cell renal cell carcinoma cohorts, GSE781 and GSE6344, datasets from the Gene Expression Omnibus (GEO) database. Multivariate survival analysis demonstrated that low expression of LRRC19 was an independent risk factor for OS. Gene set enrichment analysis (GSEA) identified tyrosine metabolism, metabolic pathways, peroxisome, and fatty acid degradation as differentially enriched with the high LRRC19 expression in KIRC cases, which are involved in selenium therapy of clear cell renal cell carcinoma. In conclusion, low expression of LRRC19 was identified as an independent risk factor, which will advance our understanding concerning the selenium adjuvant therapy of clear cell renal cell carcinoma.

## 1. Introduction

Clear cell renal cell carcinoma (ccRCC), namely kidney renal clear cell carcinoma (KIRC) in the TCGA database, is the most common malignancy of renal cancer, representing 75–82% of primary malignancies of the kidney [1,2,3]. Studies link obesity, smoking, type 2 diabetes, hypertension, and alcohol use to modifiable risk factors for KIRC [4]. Over half of new KIRC cases are found incidentally for the asymptomatic disease course, which continues to represent major a barrier in the treatment of advanced KIRCs and make it lethal [5]. KIRC in situ is usually treated with a partial or radical nephrectomy by surgery, with tumor ablation or active monitoring of small tumors. For metastatic patients, systemic therapy, including immunotherapy, is primarily reserved. However, drug resistance and dose-limiting toxicities still kill patients with advanced cancer, which calls for the identifications of novel treatment, especially for the advanced KIRCs.

For decades, people have seen the progress of KIRC treatment, such as agents of anti-angiogenesis, immune checkpoint inhibitors, and other biologically driven target therapies. Even though the majority of KIRCs are characterized by a clear cytoplasm with extensive lipid content [6], the molecular basis of KIRC is extremely complex, and several molecular markers have been proposed [2]. Regulation of hypoxia-inducible factors (HIF) pathway by Von Hippel–Lindau (VHL) gene under hypoxic conditions [7,8,9,10], vascular endothelial growth factor (VEGF) pathway tyrosine kinase inhibitors (TKIs) for anti-angiogenesis, rapamycin (mTOR) inhibitors, specific oncogenic miRNA alterations, and anti-programmed death 1 (PD-1)/programmed death ligand 1 (PD-L1) therapies are promising therapeutic agents that have improved the treatment landscape.

Selenium (Se) is a necessary trace element for humans and animals, which can be supplied from diet. Its biological function is extensively involved in the regulation of cell metabolism, especially at the active site of several functional proteins of the antioxidant network [11,12]. The anti-tumor effects of selenium have been reported previously, e.g. selenium may reduce the treatment-induced toxicities, allowing for 2–3 times of the tolerated doses of irinotecan [13]. At the molecular level, selenium mainly plays a biological role in the form of 25 selenoproteins in the human body. The amount of selenoprotein can equal the human selenium level to some extent, in addition to selenomethionine, a major pool of selenium in the human body [14]. In selenoproteins, the major active form of selenium is selenocysteine (Sec), which was identified as the 21st amino acid. Sec is biosynthesized and incorporated into selenoproteins, which plays a significant role in biological processes, such as anti-carcinogenesis. Supplemental Se enables the full endogenous response of selenoprotein expression [15].

In many epidemiological studies, researchers mainly focus on selenium status in the human body, selenium intake in the diet, selenium intake in various forms, etc. These studies were based on the theory that selenium can directly affect the biosynthesis of Sec, which affects the translation of selenoprotein genes in the human body. However, they overlap with the gene expression of selenoproteins, especially at the transcript level. To date, studies of selenoproteins raised on the association between Se supplements and cancer prevention, as well as GPX1, GPX2, and GPX3, have been reported with major physiological roles of cancer prevention or development [16]. Unfortunately, research on selenoproteins and cancer is still in its infancy, and there are very few reports of more in-depth related mechanisms. Applying high-throughput data sources can help researchers quickly screen out key genes related to selenoprotein function, and illustrate novel targets for the molecular biological mechanisms of selenium in cancer therapy.

**Figure 1:**
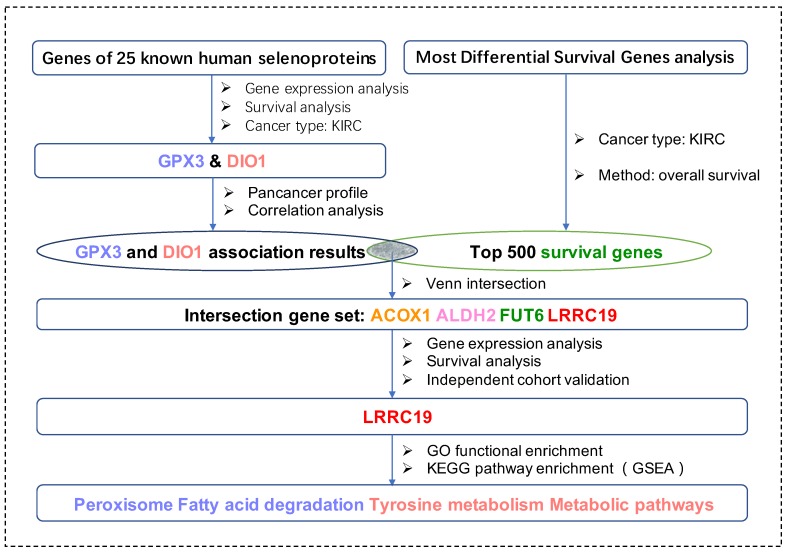
The workflow of the current work. Abbreviations are defined as follows: kidney renal clear cell carcinoma (KIRC), leucine-rich repeat-containing protein 19 (LRRC19), aldehyde dehydrogenase 2 family member (ALDH2), fucosyltransferase 6 (FUT6) and acyl-CoA oxidase 1 (ACOX1), Kyoto Encyclopedia of Genes and Genomes (KEGG), Gene Ontology (GO), Gene Set Enrichment Analysis (GSEA).

For the first time in this current work, we applied bioinformatic methods on KIRCs and normal cases from the TCGA database and GTEx database with the aim to identify key genes (DIO1 and GPX3) from the total 25 selenoproteins. We took advantage of survival maps to investigate prognostic selenoproteins with significant survival probability in KIRC and selected the ones with differential expression between KIRCs and normal cases. Moreover, we matched DIO1 and GPX3 associated genes with the most common survival genes of KIRC. The four matched most common survival genes, namely leucine-rich repeat-containing protein 19 (LRRC19), aldehyde dehydrogenase 2 family member (ALDH2), fucosyltransferase 6 (FUT6), and acyl-CoA oxidase 1 (ACOX1), were further screened in the KIRC dataset with both differential expression and survival curves, and only LRRC19 were screened out. Enrichment analysis of LRRC19 showed potential mechanisms of its correlations to GPX3 and DIO1. Importantly, we validated such correlation in two independent KIRC cohorts available from the GEO database. In particular, this protocol is universal with all TCGA cancer types, suited for cancer research of selenium. Figure 1 shows the workflow of our study.

## 2. Materials and Methods

### 2.1. Database

The gene expression profile and clinical data for renal clear cell carcinoma patients were obtained from the KIRC dataset (523 tumors and 72 normal) from TCGA (URL: https://portal.gdc.cancer.gov/). The Genotype-Tissue Expression (GTEx) [17] project provides an analysis of RNA sequencing data from 1641 samples across 43 tissues from 175 individuals. Gene Expression Profiling and Interactive Analyses vision 2 (GEPIA2) (URL: http://gepia2.cancer-pku.cn/) is a web server for analyzing the RNA sequencing expression data of 9736 tumors and 8587 normal samples from the TCGA and the GTEx projects, using a standard processing pipeline [18]. We obtained access to GTEx data and analyzed the kidney dataset (28 cases) as part of the normal control group by GEPIA2. The Gene Expression Omnibus (GEO) database (URL: https://www.ncbi.nlm.nih.gov/geo/) is an open-access platform for microarray data. The relevant microarray dataset was analyzed with the online tool GEO2R of GEO to identify genes that were differentially expressed across experimental conditions.

### 2.2. Differential Expression Analysis

The dot plots of the pan-cancer gene expression profile, the box plots of differential expression genes, and the violin plot of the patient pathological stage were analyzed and drawn in GEPIA2. The KIRC cases were paired with normal samples from TCGA. The expression data were first $\log_{2}\left(TPM+1\right)$ transformed and the value of $\log_{2}FoldChange\left(FC\right)$ was defined as median (Tumor) vs. median (Normal). Genes with $|\log_{2}FC|>1$ and adjust.p-value < 0.01 were considered as differentially expressed genes (DEGs). The method for stage plot was one-way ANOVA, using the pathological stage as a variable for calculating differential expression. Pr(>F) < 0.05 was considered statistically significant.

UALCAN (URL: http://ualcan.path.uab.edu/) is a comprehensive, user-friendly, and interactive web resource for analyzing cancer omics data (including the TCGA data) [19]. We performed an analysis of expression levels of relative key genes among the KIRC sub-groups, based on individual gender, age, race, grade, and nodal metastasis status, using UCLCAN.

GEO2R was applied to compare the mRNA differential expression levels of key genes between renal clear cell group and normal groups to validate the key genes that are identified from TCGA. We downloaded mRNA profiling of renal clear cell relevant series, GSE781 [20] and GSE6344 [21,22], at GEO. These RNA profiles were performed on the GPL96 platform. The results are presented as a bar plot showing the logFoldChange of gene expression.

### 2.3. Survival Analysis

Survival analysis, survival map, and the list of most differential survival genes were generated from GEPIA2. Kaplan–Meier plots (K-M plots) showed overall survival (OS) or disease-free survival (DFS, also called relapse-free survival (RFS)) analysis based on gene expression; the median was selected as the threshold for splitting the high-expression and low-expression cohorts (Cutoff = 50%); the hazards ratio (HR) based on Cox PH Model were calculated; *p*-value < 0.05 was considered as statistically significant using the log-rank test. The survival map is a heat map, showing the survival analysis results of gene lists across a pan-cancer scale. OS and DFS method were performed, using months as the survival time units; high and low groups were cutoff with median; and *p*-value (no adjustment) < 0.05 was set as the significance level. The most differential survival genes were drawn as a table of top 500 genes most associated with KIRC patient overall survival; the expression threshold for splitting high-expression and low-expression cohorts was cut off by median.

Univariate and multivariable Cox analysis were performed to verify the correlations between LRRC19 expression and survival along with important covariates such as patient’s age and gender, tumor’s grade, and other clinical factors that are widely available for the TCGA cohort. *p*-value < 0.05 was considered statistically significant. The data were processed using R software (version 3.6.1) and Strawberry Perl (version 5.30).

### 2.4. Correlation Analysis

LinkedOmics (URL: http://www.linkedomics.org/login.php) [23] is an open-access web-based portal that analyzes and compares cancer multi-omics data within and across all 32 TCGA cancer types. It is also multi-dimensional that includes mass spectrometry-based proteomics data generated by the Clinical Proteomics Tumor Analysis Consortium (CPTAC) for TCGA breast, colorectal, and ovarian tumors. The *LinkFinder* module of LinkedOmics was used to search for differential expression genes in correlation with GPX3 and DIO1 in the KIRC dataset on the Hi-seq RNA platform (533 patients). The *LinkFinder* also visualized analysis results by volcano plots, heat maps, and scatter plots for individual genes. The results were analyzed using Pearson’s correlation coefficient (*R*); |*R*| > 0.3 and false discovery rate (FDR) < 0.05 were considered as statistically significant. The correlation analysis was performed in the datasets of “523 TCGA tumor cases + 72 TCGA normal cases” and “523 TCGA tumor cases + 72 TCGA normal cases + 28 GTEx Kidney—Cortex cases” to improve the results, by GEPIA2. The results were analyzed using Pearson’s correlation coefficient (*R*); |*R*| > 0.3 and *p* < 0.05 were considered as statistically significant.

### 2.5. Venn Diagram

We used the online tool Draw Venn Diagram (URL: http://bioinformatics.psb.ugent.be/webtools/Venn/) to calculate the intersections of top 500 most common survival genes and differential expression genes in correlation with GPX3 and DIO1 in KIRC. The textual and graphical outputs were generated for screening prognostic genes having correlations with both GPX3 and DIO1.

### 2.6. Enrichment Analysis

The *Link-Interpreter* module of LinkedOmics performs enrichment analysis of LRRC19 associated genes. Link-Interpreter module transforms association results generated by LinkFinder into biological understanding, based on Kyoto Encyclopedia of Genes and Genomes (KEGG) pathway and Gene Ontology (GO) database. The Web-based Gene SeT AnaLysis Toolkit (WebGestalt) [24,25,26] provided the comprehensive functional category database. It is designed to continuously generate the function of genomic, proteomic, and large-scale genetic studies of big datasets, such as differentially expressed gene sets, co-expressed gene sets, etc. WebGestalt incorporates information from different public resources and provides an easy way for biologists to make sense out of gene lists. In the *Link-Interpreter* module, the data from *LinkFinder* result were signed and ranked by FDR, and Gene Set Enrichment Analysis (GSEA) [27] was used to generate analyses of GO function (Biological Process, Cellular Component, and Molecular Function) and KEGG pathway. The minimum number within per gene size was set as 10, and 500 simulations were performed.

## 3. Results

### 3.1. Identification of KIRC-Related Selenoproteins

Twenty-five selenoprotein genes were set as the input and screened with prognostic value and gene expression level in KIRC. We drew survival maps of the 25 selenoproteins in KIRC cases from TCGA. Both of the overall survival and disease-free survival were analyzed, and heat maps of hazard ratio (HR) were drawn to show the prognostic value of individual selenoprotetin (Appendix A). Nine selenoproteins [28], namely SELENOP (selenoprotein P, SeP, SEPP1, SELP), SELENON (selenoprotein N, SEPN1, SELN), SEPHS2 (selenophosphate synthetase 2), SELENOM (selenoprotein M, SELM), SELENOI (selenoprotein I, SELI, EPT1), SELENOT (selenoprotein T, SELT), SELENOF (selenoprotein F, the 15-kDa selenoprotein, SEP15), GPX3, and DIO1, showed a significant difference on overall survival and/or disease-free survival in KIRC cases. Then, we analyzed the differential gene expression levels of the nine prognostic selenoproteins by comparing the transcription levels in KIRCs and normal cases. Gene expressions in KIRC samples that were changed by more than two-fold were defined as meaningful, thus only GPX3 and DIO1 were screened.

**Figure 2:**
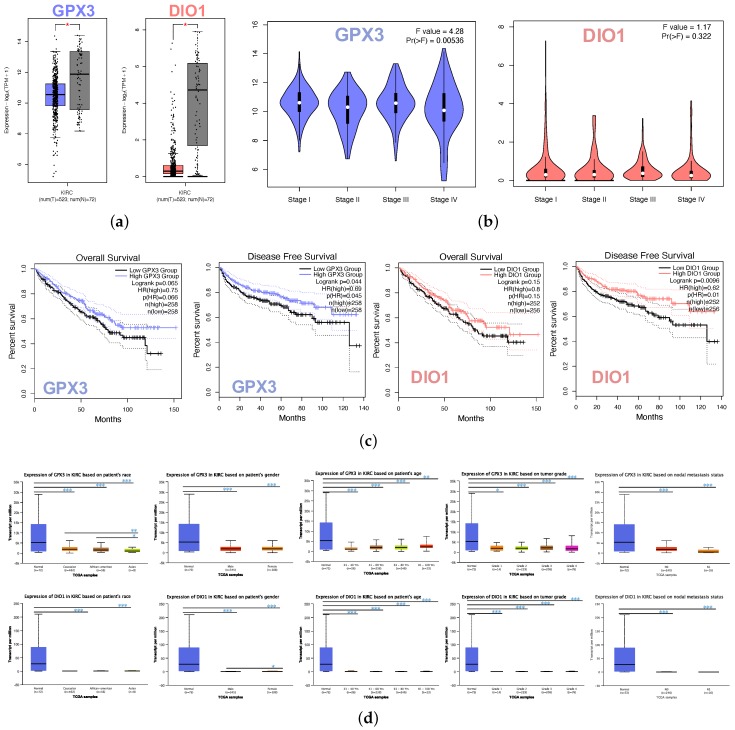
Identifications of significant KIRC-related selenoproteins. (**a**) Different expression of GPX3 and DIO1 in KIRC samples and healthy samples by box plot. tumor group (T), normal group (N). *p* value < 0.05. (**b**) Correlation between GPX3 and DIO1 expression and KIRC tumor stage in the violin plot. The expression of GPX3 was significantly downregulated in Stage IV. Pr(>F)=0.00536. (**c**) KIRC cases were divided into two groups based on their individual expression levels. As shown in the Kaplan–Meier survival curve, median disease free survival of the high expression groups is longer than the low expression group significantly, as indicated by the log-rank test, *p* value < 0.05. However, the median overall survival is not statistically different as indicated by the log-rank test, *p* value > 0.05. (**d**) Box plots showing relative transcription of GPX3 and DIO1 in subgroups of KIRC patients, stratified based on gender, age, race, tumor grade, and metastasis status. Data are mean ± SE. * *p* < 0.05; ** *p* < 0.01; *** *p* < 0.001.

The box plot indicated the lower expression levels of GPX3 and DIO1 in KIRC cases than normal cases (Figure 2a). We also analyzed the expression of GPX3 and DIO1 with the KIRC tumor stage. GPX3 was significantly downregulated in Stage IV, whereas DIO1 did not significantly vary (Figure 2b). We further explored the critical efficiency of GPX3 and DIO1 in the survival of KIRC cases by Kaplan–Meier plots (K-M plots). Survival curves were performed to analyze the association between the mRNA expression and the survival probability of KIRC patients. The survival curve and log-rank test analyses revealed that the decreased GPX3 (*p* < 0.05) and DIO1 (*p* < 0.01) levels were significantly correlated with the disease-free survival (DFS); however, the overall survival (OS) showed no significance (Figure 2c). The KIRC patients with higher mRNA levels of GPX3 and DIO1 were predicted to have good prognoses.

Further sub-type analysis of multiple clinic pathological traits of 533 KIRC samples in the TCGA database consistently showed low expression of GPX3 and DIO1 (UALCAN). The transcription levels of GPX3 and DIO1 were significantly lower in KIRC patients than normal control cases in subgroup analysis based on gender, age, tumor grade, and nodal metastasis status (Figure 2d). Thus, GPX3 and DIO1 expression may serve a potential diagnostic indicator in KIRC, representing a potential mechanism of sufficient selenium status in the human body protecting against renal clear cell carcinoma genesis.

### 3.2. GPX3 and DIO1 Gene Expression in Pan-Cancer

**Figure 3:**
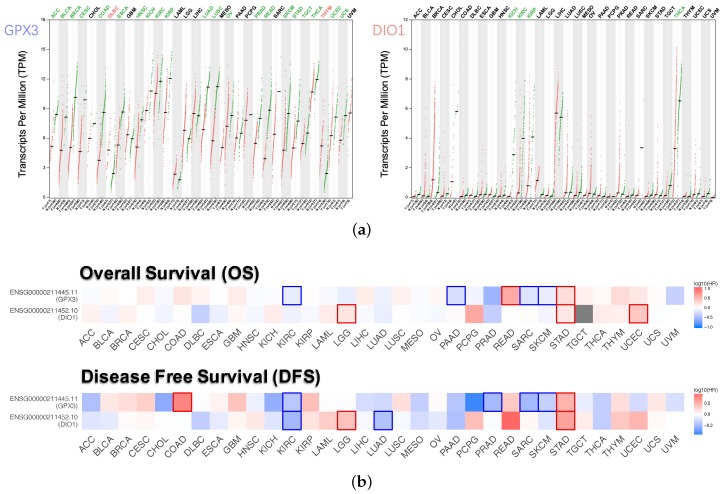
Expression level and survival analysis of GPX3 and DIO1 across 33 TCGA cancer types (GEPIA2). (**a**) Dot plots of GPX3 and DIO1 differential expression levels in 33 cancer types (TCGA) compared to the normal (TCGA normal and GTEx). Each dot represents a distinct tumor (pink) or normal sample (green). The abscissa represents the sample from tumor tissue (T) or normal tissue (N), and “n” represents the sample size. The ordinate represents the amount of transcript expression in the sample, and the expression data were $\log_{2}\left(TPM+1\right)$ transformed. Above the table are the TCGA abbreviations of the cancer names. The cancer name coding in green indicates that the target gene has relatively lower expression in the tumor tissue, and the red indicates higher expression. We selected LIMMA as the differential method. |$\log_{2}FC$| > 1 and FDR < 0.01 were considered as differentially expressed. (**b**) Heat map of hazard ratios (HR) illustrating cancer-GPX3/DIO1 pairs with altered prognosis. We selected the median as the suitable expression threshold for splitting the high-expression and low-expression cohorts. The hazards ratio (HR) was calculated based on the Cox PH Model, and the HR value was scaled in decibel (dB). The red and blue color presented high and low risk, respectively, and the intensity of color indicated the value of HR. The bounding box around the tiles represented the statistically significant cancer types (*p* < 0.05). Adrenocortical Carcinoma (ACC), Bladder Urothelial Carcinoma (BLCA), Breast Invasive Carcinoma (BRCA), Cervical Squamous Cell Carcinoma and Endocervical Adenocarcinoma (CESC), Cholangiocarcinoma (CHOL), Colon Adenocarcinoma (COAD), Lymphoid Neoplasm Diffuse Large B-cell Lymphoma (DLBC), Esophageal Carcinoma (ESCA), Head and Neck Squamous Cell Carcinoma (HNSC), Kidney Chromophobe (KICH), Kidney Renal Clear Cell Carcinoma (KIRC), Kidney Renal Papillary Cell Carcinoma (KIRP), Acute Myeloid Leukemia (LAML), Brain Lower Grade Glioma (LGG), Liver Hepatocellular Carcinoma (LIHC), Lung Adenocarcinoma (LUAD), Lung Squamous Cell Carcinoma (LUSC), Mesothelioma (MESO), Ovarian Serous Cystadenocarcinoma (OV), Pancreatic Adenocarcinoma (PAAD), Pheochromocytoma and Paraganglioma (PCPG), Prostate Adenocarcinoma (PRAD), Rectum Adenocarcinoma (READ), Sarcoma (SARC), Skin Cutaneous Melanoma (SKCM), Stomach Adenocarcinoma (STAD), Testicular Germ Cell Tumors (TGCT), Thyroid carcinoma (THCA), Thymoma (THYM), Uterine Corpus Endometrial Carcinoma (UCEC), Uterine Carcinosarcoma (UCS), Uveal Melanoma (UVM).

Dot plots (Figure 3a) described the differential gene expression levels with respect to GPX3 and DIO1 among the 33 TCGA cancer types. The tumor cases were collected from the TCGA database. The normal samples were obtained from both TCGA normal datasets and healthy kidney cortex tissues from the GTEx database. It is observed that DIO1 was downregulated in four cancer types: Kidney Chromophobe (KICH), Kidney Renal Clear Cell Carcinoma (KIRC), Kidney Renal Papillary Cell Carcinoma (KIRP), and Thyroid carcinoma (THCA). In addition, we found that GPX3 expression was reduced in 21 types of cancers. However, the expression of GPX3 was increased in two cancer types: Lymphoid Neoplasm Diffuse Large B-cell Lymphoma (DLBC) and Thymoma (THYM). Based on the above results, we conclude that most of the tumor tissues, if not all, might be in a state of selenium deficiency.

Survival maps (Figure 3b), i.e. heat maps of hazard ratio (HR), showed the prognostic value of GPX3 and DIO1 in pan-cancer. We selected 50% as the cutoff for splitting the high-expression and low-expression groups. The HR was calculated based on the Cox PH Model, and the HR value was scaled in decibel (dB). Statistically significant (*p* < 0.05) types of cancer were framed. Considering both OS and DFS, high expression of DIO1 and GPX3 indicated improved prognosis in Brain Lower Grade Glioma (LGG) and Stomach adenocarcinoma (STAD). However, both indicated poor outcome in KIRC, Sarcoma (SARC), and Skin Cutaneous Melanoma (SKCM). In addition, for the four types of cancer, only one of GPX3 or DIO1 showed prognostic value. High expression of DIO1 indicated a good prognosis in Lung Adenocarcinoma (LUAD). High expression of GPX3 indicated good prognosis in Pancreatic Adenocarcinoma (PAAD), as well as higher risk in Rectum Adenocarcinoma (READ) and Uterine Corpus Endometrial Carcinoma (UCEC).

### 3.3. Most Common Survival Genes in Correlation with GPX3 and DIO1 in KIRC

**Figure 4:**
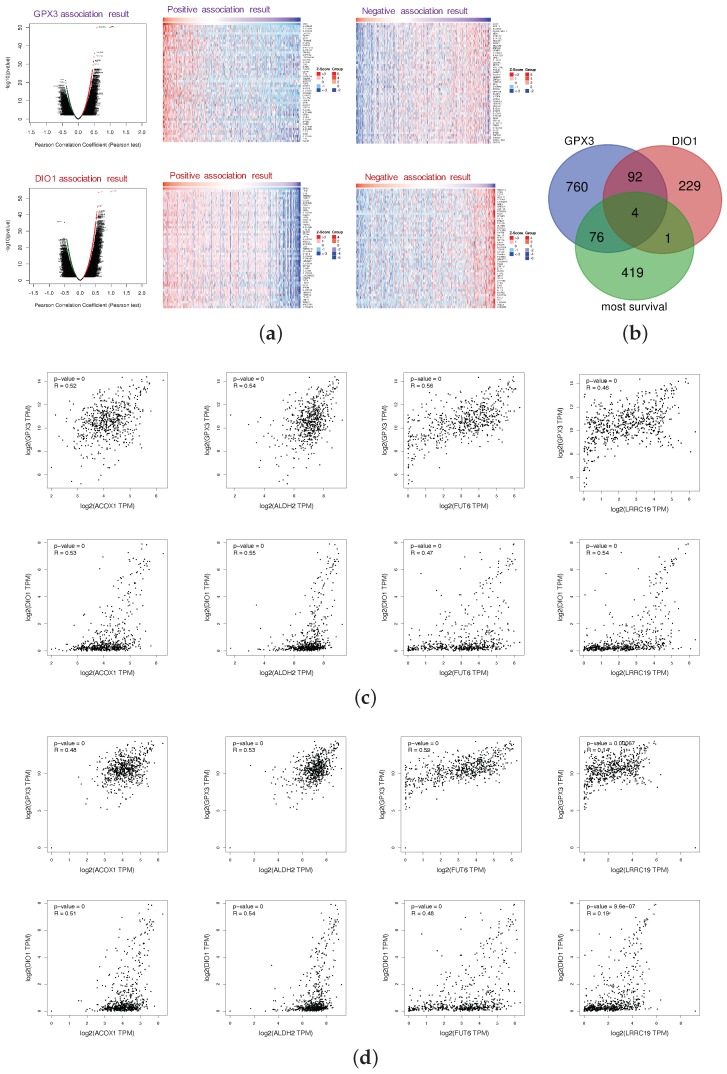
Most survival genes in correlation with GPX3 and DIO1 in KIRC. (**a**) Association results of GPX3 and DIO1 in KIRC (*n* = 533); Pearson test was performed to analyze *LinkFinder*. Volcano plots show the statistical association results of GPX3/DIO1 for all KIRC differential expression genes, and individual genes of interest can be obtained in Table S1. Data for the top 50 positive and negative association genes are visualized in heat maps, respectively. (**b**) Venn diagram shows the mapping results among KIRC most common survival genes (green), GPX3 (purple), and DIO1 (pink) associated gene lists. There were four genes, namely ACOX1, ALDH2, FUT6, and LRRC19, in the intersection of the three datasets. (**c**) Correlation results of intersection genes with GPX3 and DIO1 based on datasets of “KIRC (*n* = 523) + TCGA normal cases (*n* = 72)”, using Pearson test. (**d**) Correlation analysis of intersection genes with GPX3 and DIO1 using data from "KIRC (*n* = 523) + TCGA normal cases (*n* = 72) + GTEx kidney cortex samples (*n* = 28)", using Pearson test. The association results with Pearson correlation coefficient (*R*) > 0.3 and *p* < 0.05 were considered significant (GEPIA2).

The *LinkFinder* module of LinkedOmics was used to perform association genes of GPX3 and DIO1 based on the data of 533 KIRC patients. The platform was selected as HiSeq RNA in both the Search dataset and the Target dataset (28 January 2016). The data were normalized by the RNA-seq by expectation maximization (RSEM) pipeline. In the correlation analysis, *p*-value and coefficient were obtained from the statistical method of Pearson, which stood for the statistical significance and the degree of correlation, respectively. Since the results of the correlation analysis should consider both the significance and the value of the correlation coefficient, volcano plots (Figure 4a) were generated to overview the correlation analysis results, wherein each dot represents a gene. Overall, 651 genes (red dots) showed strong positive correlations with GPX3, whereas 282 genes (green) showed strong negative correlations; and 282 genes (red dots) showed strong positive correlations with DIO1, whereas 44 genes (green) showed strong negative correlations (FDR < 0.01, Pearson correlation coefficient *R* > 0.3). As shown in the volcano plots, GPX3 and DIO1 had more positive correlation genes than the negative ones. The individual genes of interest can be queried in Table S1. The top-rank significant genes positively and negatively associated with GPX3 and DIO1 are shown in the heat maps of Figure 4a, respectively. This results indicate a widespread impact of selenoproteins GPX3 and DIO1 at the transcriptome level.

We generated the 500 most common survival genes of KIRC by GEPIA2 (Table S2). Then, we drew Venn diagram to explore intersections among the three datasets, GPX3 association genes, DIO1 association genes, and KIRC most common survival genes (Figure 4b). There were four intersection genes, namely ACOX1, ALDH2, FUT6, and LRRC19, representing the potential correlations between selenium status and KIRC survival probability. The association results for individual genes were based on the KIRC dataset (*LinkFinder*) and are shown in Appendix B.

To explore whether GPX3, DIO1, and LRRC19 still correlate when including the normal tissue samples, we performed correlation analysis in the datasets of “523 TCGA tumor cases + 72 TCGA normal cases” (Figure 4c) and “523 TCGA tumor cases + 72 TCGA normal cases + 28 GTEx Kidney-Cortex cases” (Figure 4d). We also calculated the Pearson’s correlation coefficient for statistical tests. *R* > 0.3 and *p* < 0.05 were considered statistically significant. Based on the expanded correlation analysis results, we found that ACOX1, ALDH2, FUT6, and LRRC19 have a high association with GPX3 and DIO1 in the TCGA database, whether it is only tumor samples (Appendix C) or “tumor samples + normal samples” (Figure 4c). Adding another independent healthy kidney tissue cohort from GTEx (Figure 4d), ACOX1, ALDH2, and FUT6 still have a relatively high correlation, while the correlation of LRRC19 decreased sharply. It is extremely interesting that the correlation analysis of LRRC19 dramatically differed depending on whether it took the GTEx cohort into account. Our analysis assumes that KIRC patients were generally in a low selenium state, and the gene expression of LRRC19 in KIRCs and adjacent tissues might both be affected by selenium deficiency to some extent. However, GTEx samples were collected from a healthy population, which may reduce the correlation between LRRC19 and selenoprotein, due to the optimal selenium levels of the healthy.

### 3.4. Screen and Validation of Intersection Genes

**Figure 5:**
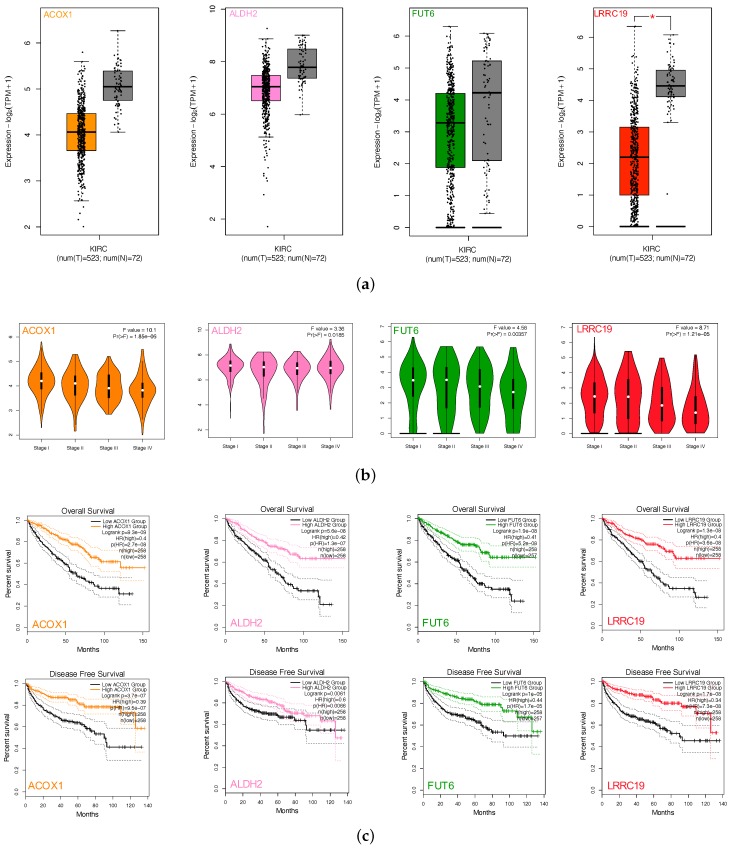

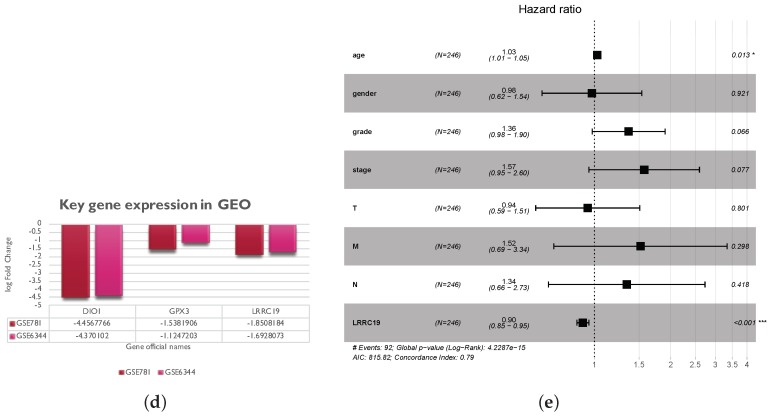
Screen and validation of intersection genes. (**a**) Validation of ACOX1, ALDH2, FUT6, and LRRC19 differential expression in KICR on GEPIA2. Box plots show that there was a significant differential expression of LRRC19 between KIRC cases and normal cases (|$\log_{2}FC$| > 1 and *p* < 0.05). (**b**) Stage plots of intersection genes expression with tumor stage in KICR on GEPIA2. Violin plots show ACOX1, FUT6, and LRRC19 were significantly downregulated in Stage IV (*p* < 0.001). (**c**) Kaplan–Meier plots show the prognostic value of ACOX1, ALDH2, FUT6, and LRRC19. Overall and disease-free survival curves were generated for the comparison of survival percent between groups with high and low gene expression (*p* < 0.01 in Log-rank test). (**d**) Validation of DIO1, GPX3, and LRRC19 expression fold change in independent GEO cohorts, GSE781 and GSE6344. (**e**) Multivariate analysis of the correlation of LRRC19 expression with OS among KIRC patients. The columns in the forest plot are parameter, number of patients, HR (IC 95%), and *p* value. (* *p* < 0.05, ** *p* < 0.01, *** *p* < 0.001).

We performed gene expression analysis and survival curves of the four intersection genes (ACOX1, ALDH2, FUT6, and LRRC19) by GEPIA2. Box plots (Figure 5a) showed differential gene expressions of the four genes between KIRCs and normal cases (TCGA). All four gene expression levels decreased compared with the normal samples. However, LRRC19 was the only one that showed statistically significant change of two folds (*p*-value < 0.05 and |$\log_{2}FC$| > 1). Violin plots (Figure 5b) showed the expression levels of the four genes with the tumor stages of KIRCs. ACOX1, FUT6, and LRRC19 expression levels dramatically decreased in Stage IV, whereas ALDH2 did not vary (*p*-value < 0.01). K-M plots (Figure 5c) revealed the low risk of ACOX1, ALDH2, FUT6, and LRRC19 in KIRC with both overall survival and disease-free survival (*p*-value < 0.01). Taking the significantly differential expression and prognostic value in KIRC together, we selected LRRC19 as the key genes of our work for further analysis. As the only intersection gene with significant differential expression and prognostic value, we also performed the survival analysis and gene expression of LRRC19 in 33 cancer types of TCGA (Appendix B). From the results, LRRC19 showed prognostic value in Cholangiocarcinoma (CHOL), Colon Adenocarcinoma (COAD), Kidney Renal Clear Cell Carcinoma (KIRC), Kidney Renal Papillary Cell Carcinoma (KIRP), Liver Hepatocellular Carcinoma (LIHC), Lung Adenocarcinoma (LUAD), Ovarian Serous Cystadenocarcinoma (OV), and Rectum Adenocarcinoma (READ). The differential expression of LRRC19 was observed to be downregulated in Kidney Chromophobe (KICH) and KIRC and upregulated in Acute Myeloid Leukemia (LAML).

To further validate the key genes (GPX3, DIO1, and LRRC19) identified from the TCGA and GTEx database, we analyzed gene expression data of the key genes in two other individual cohorts. The data (GSE781 and GSE6344) were downloaded from GEO, an independent microarray database of renal clear cell carcinoma. All three genes were significantly downregulated in both datasets (Figure 5d).

The effect of covariates in the survival analysis might lead to biases in the results and misinterpretations, and multivariate survival analysis would be important to confirm the correctness of the discovery. We also did survival analysis of LRRC19, taking into account important covariates such as patient’s age and gender, tumor’s grade, and other clinical factors available for the TCGA cohort. The input data and the results of univariate and multivariate Cox analyses are shown in Table S3. The forest plot (Figure 5e) showed that low LRRC19 expression was an independent risk factor for OS among KIRCs (hazard ratio [HR] = 0.9, 95% confidence interval [CI] = 0.85-0.95, *p* = 0.000144).

### 3.5. Enrichment Analysis of LRRC19 Association Genes

**Figure 6:**
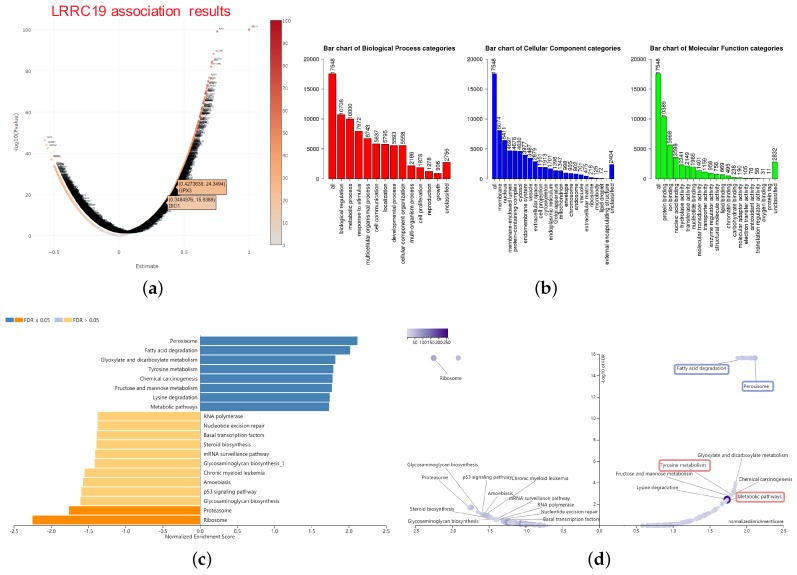
Significant enrichment plots of LRRC19, using gene set enrichment analysis (GSEA). (**a**) Genes differentially expressed in correlation with LRRC19 in KIRCs. The positions of the dots representing GPX3 and DIO1 are marked. (**b**) Gene Ontology (GO) Slim summary for the LRRC19 association results. Each biological process (BP), cellular component (CC), and molecular function (MF) category is represented by a red, blue, and green bar, respectively. The height of the bar represents the number of IDs in the user list as well as in the category. The significantly enriched Kyoto Encyclopedia of Genes and Genomes (KEGG) pathways of LRRC19 co-expression genes in KIRC were analyzed by GSEA. The enriched KEGG pathway result was clustered with affinity propagation for redundancy reduction, and clustered pathways are shown. (**c**) In the bar plots, the color scale represents the FDR value, and the length of the bar represents the normalized enrichment score (NES) value (positive correlated, blue > 0; and negative correlated, orange < 0). (**d**) In the volcano plots, the intensity of color and jitter size indicates the number of the elements in each pathway. The four pathways of interest are framed; purple indicates GPX3 correlation and pink indicates DIO1 correlation.

The LRRC19 association results were analyzed and visualized in the volcano plot (Figure 6a), using the *LinkInterpreter* module of LinkedOmics. In the *LinkInterpreter* module, we performed the method of gene set enrichment analysis (GSEA) [27] to unambiguously map LRRC19 association gene set with categories defined by Gene Ontology (GO) term and pathways from Kyoto Encyclopedia of Genes and Genomes (KEGG), through accessing the comprehensive functional category database in WebGestalt (http://www.webgestalt.org) [25,26].

Bar plot (Figure 6b) showed a slim summary of significant GO term analysis. The result indicated that genes differentially expressed in correlation with LRRC19 were located mainly in the biological regulation, metabolic process, and response to the stimulus of the biological process (BP) categories; membrane, nucleus, and membrane-enclosed lumen of the cellular component (CC) categories; and protein binding of molecular function (MF) categories, respectively. These categories showed participation primarily in biological metabolism, signaling cascade, and signal transduction pathway and matched the cellular location of LRRC19 as a membrane protein.

The enriched KEGG pathway result was clustered with affinity propagation for redundancy reduction, using the R package “apcluster”. Then, clustered KEGG terms were visualized by bar plot and scatter plot (Figure 6c).

### 3.6. GPX3 and DIO1 Associated Pathways

**Figure 7:**
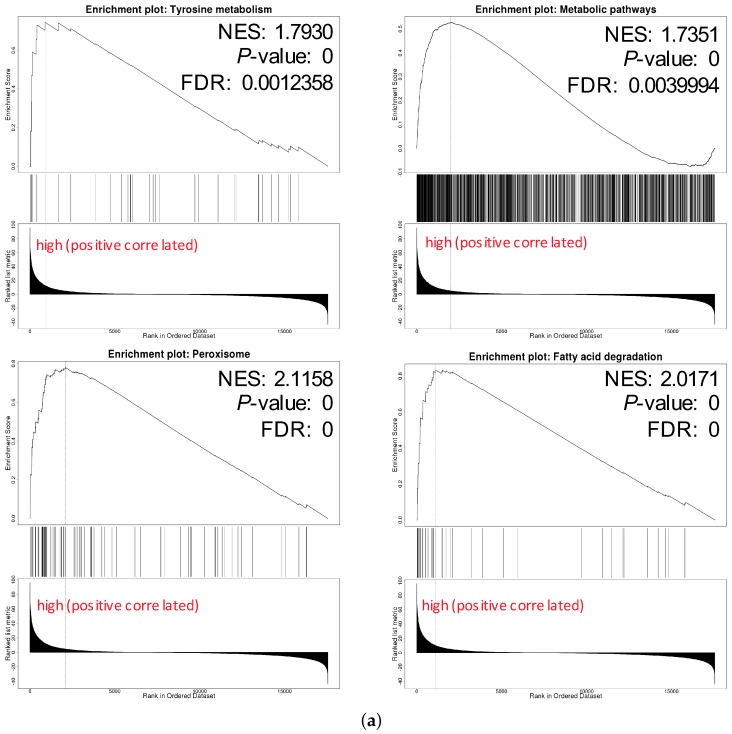

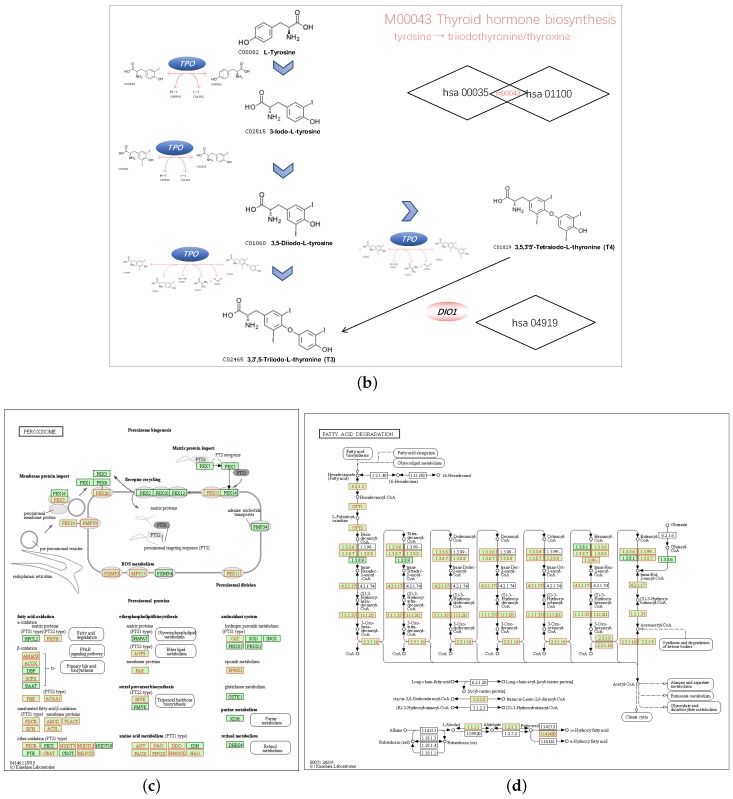
Pathways of interest. (**a**) GSEA results showing tyrosine metabolism, metabolic pathways, peroxisome and fatty acid degradation are differentially enriched pathways in LRRC19-related genes. NES, normalized enrichment score. (**b**) The relationship between DIO1 and Module 00043 Thyroid hormone biosynthesis. (**c**) KEGG pathway annotations of the peroxisome pathway. (**d**) KEGG pathway annotations of the fatty acid degradation pathway. Altered genes were denoted in red.

We chose hsa04146 Peroxisome and hsa00071 Fatty acid degradation for their highest normalized enrichment score (NES) and FDR value. They also represented the potential to correlate to the biological progress of GPX3, compared with the KEGG pathway term of the Ribosome. The hsa00035 Tyrosine metabolism and hsa01100 Metabolic pathways were also selected as the interesting terms and the GSEA results are shown in detail in Figure 7a. Tyrosine metabolism and Metabolic pathways were closely associated with the function of thyroid hormones regulated by DIO1. In the results of the enrichment analysis, hsa00035 Tyrosine metabolism and hsa01100 Metabolic pathways had high NES scores, which were 1.7930 and 1.7351, respectively. Among them, Module 00043 Thyroid hormone biosynthesis was the intersection of these two pathways, which represents the transformation process of tyrosine to triiodothyronine (T3)/thyroxine (T4). Referring to hsa04919 Thyroid hormone signaling pathway, we determined the relationship between DIO1 and M00043 (Figure 7b). Peroxisome (Figure 7c) and Fatty acid degradation (Figure 7d) were the top two terms ranked with the normalized enrichment score (NES).

## 4. Discussion

Past years have seen a dramatic improvement in treatment methods for KIRC, which has shed light on the significance of identifying biomarkers that contribute to the tumorigenesis of KIRC to provide potential targets for KIRC therapy. Antagonistic associations between selenium and cancer have been widely reported in previous studies. For example, selenomethionine (SLM) and Se-methylselenocysteine (MSC) are organic forms of selenium, converted by plants from inorganic forms of selenium, such as selenide and selenite. SLM and MSC are currently being studied as anti-cancer compounds, and SLM has been approved for clinical trials by FDA [5]. The active metabolite of MSC is methylselenic acid (MSA), which was found to inhibit HIF1α expression and activity in the setting of hypoxia [29]. β-catenin was also found to be a target for selenium in the reduction of β-catenin correlated drug-resistance [30]. Angiogenesis and drug-resistance associated miRNAs, such as hsa-miR-155 and hsa-miR-210, may also be regulated by selenium compounds [6,31,32]. Increasing evidence suggests that selenium can affect immune checkpoint PD-1/PD-L1 by regulating HIFs and oncogenic miRNA-155 and miRNA-210, transcriptionally and post-transcriptionally, under hypoxic conditions [6,33,34,35].

Although the relationship between selenium and tumors has been widely reported in epidemiological studies [36,37,38,39,40], the evidence of taking selenium as an anticancer drug alone seems to be insufficient. Reports of MSC have described the anti-tumor effect associated with irinotecan through the affection of the tumor microenvironment (TME), including the inhibition of angiogenesis [29].

In these studies, selenium has a sufficient geographic scientific basis as an adjuvant treatment of cancer [6], reducing the toxicity of chemotherapeutic drugs, increasing the subject’s tolerance to chemotherapeutic drugs, etc. However, these studies have been conducted to detect cancer-related target genes in various dosage forms of selenium only, and all of them ignored the detection of selenoprotein gene expression and the uncovered mechanisms that selenoproteins play the biological functions. Interestingly, even studies focusing on the relationship between selenoproteins and cancer in vivo rarely are performed in the human body. It should be emphasized that the relationship between selenium and cancer must not only consider selenium intake and dosage form. For a cancer patient, mutations of the selenoprotein gene and regulation of selenoprotein gene expression may affect the effects on selenium adjuvant chemoradiotherapy. The kidney is an organ where selenium is excreted from the body, and thus the expression of selenoprotein may play a special role in renal clear cell carcinoma.

Existing research proves that the biosynthesis of GPX3 is tissue-specific, and it is mainly produced by the kidney and secreted into the serum to play its role [41]. In gastric cancer, decreased expression of GPX3 was reported as s a poor prognosticator [42], and GPX3 expression is mediated by genetic and epigenetic alterations caused by promoter hypermethylation [43]. In KIRCs, GPX3 was found downregulated in all 12 cases of surgically removed tumors compared with a surrounding rim of normal tissues in a Ukrainian cohort [44], and GPX3 methylation and downregulation were detected in five out of six KIRC cell lines in Liu’s research [45].

DIO1 is involved in the process of the deiodination of 3,5,3${}^{\prime }$,5${}^{\prime }$-tetraiodo-L-thyronine (thyroxine, T4) transforming into the active form of 3,5,3${}^{\prime }$-triiodothyronine (T3), a powerful regulation factor of cell differentiation, proliferation, and metabolism [46]. Previous work indicated that decrease expression of DIO1 in renal cancer resulted in altered expression of genes related to cell cycle progression, adhesion, and migration, with marked influence on proliferation and cell motility [47,48,49], and the regulation of DIO1 gene expression is relatively well understood [50,51,52,53]. Selenium compounds in a series, especially selenium-enriched yeast, have been clinically used to prevent or treat benign and malignant thyroid diseases such as goiter, autoimmune thyroid disease, or thyroid carcinoma [54,55,56]. Moreover, cases of women suffering from benign thyroid disorders, such as myxedema or lower degree thyrotoxicosis, were reported to have an increased risk for renal cancer [57].

LRRC19, a transmembrane receptor, is a member of the leucine-rich repeat (LRR)_only group [58,59]. It has a close evolutionary relationship with Toll-like receptors (TLRs) [60], a protein family identified as important components of innate immunity [61]. Chai et al. proposed that LRRC19 can activate NF-κB and induce the expression of pro-inflammatory cytokines with or without the stimulus of TLR ligands and is involved in the response to local inflammation [60]. They also demonstrated the predominant expression of LRRC19 in the kidney, where renal tubular cells play a critical regulatory role in immune-mediated diseases. Yang and colleagues declaimed the mechanisms of LRRC19 involved in the kidney’s defense against uropathogenic Escherichia coli (UPEC) infection through TRAF2/6-mediated NF-kB and MAPK signaling pathways [62]. Yang et al. also reported deficiency of LRRC19 impaired the gut immune system and decreased inflammatory responses in gut tissues [63]. LRRC19 was also reported for its therapeutic potential for pressure ulcers, by promoting NF-κB dependent pro-inflammatory response [64].

The loss of chromosomal region 9p24.1-p13.3 is implicated in metastatic clear cell renal cell carcinoma, and LRRC19 was significantly more downregulated in primary tumors with gene copy number loss than in those without it. However, LRRC19 did not affect the proliferation and apoptosis of the KIRC cell lines [65]. Manuel H. et al. reported the LRRC19 gene expression > insulin-like growth factor binding protein 2 (IGFBP2) gene expression as a biomarker for pancreatic cancer sensitivity to AZD0530, an orally active small molecule Src inhibitor [66]. They further performed a biomarker study for LRRC19 > IGFBP2, with a total of 47 patient tumor tissues from 10 different sites. However, conclusions cannot be draw on the LRRC19 > IGFBP2 classifier as a predictor, as the frequency of biomarker-positive patients was very low (<3%) [67].

In the current work, we systematically and comprehensively characterized all 25 selenoproteins in 523 renal clear cell carcinoma samples from the TCGA database and 100 normal kidney cases from the TCGA and GTEx database. Compared with previous studies, the advantage of our work is that the KIRC cohort analyzed in our work have a larger sample size, and, remarkably, we screened GPX3 and DIO1 from the total 25 selenoproteins instead of simple selenoprotein families. We found that GPX3 and DIO1 gene expression levels were significantly downregulated in KIRC cases, and the lower expression levels were associated with unfavorable clinical prognosis. This result is also corroborated with previous related research. However, the survival analysis did not take into account detailed covariates, such as age and gender, that are widely available for the TCGA cohort [68], especially the cohort with lipid metabolism and thyroid dysfunction. Moreover, we discovered a novel biomarker, LRRC19, one of the most survival genes in KIRC. LRRC19 has been reported to be involved in kidney and skin inflammatory response and as a potential biomarker in KIRC and pancreatic cancer. However, we first declaim that LRRC19 has significant correlations with key selenoproteins. Univariate and multivariable Cox analysis were performed to verify the role of LRRC19 expression as an independent prognostic factor in the OS of KIRC. Additionally, KEGG pathway enrichment analysis results of LRRC19 indicated its correlation with GPX3 and DIO1, which also implicated its function in lipid metabolism.

In the results of the enrichment analysis, Module 00043 Thyroid hormone biosynthesis was the intersection of these two DIO1 related pathways, which represents the transformation process of tyrosine to triiodothyronine (T3)/thyroxine (T4). Combining the results of hsa04919 Thyroid hormone signaling pathway, we determined the relationship between DIO1 and M00043. As a result, we speculate that LRRC19 may play a role in KIRC through the biosynthesis and activation of thyroid hormones.

KIRCs are characterized by dense lipid accumulation in morphology [6]. Obese patients with hyper-triglyceridemia have a reduced level of fatty acid oxidation, and ectopic fat accumulation may be increased oxidation of fatty acids [69,70]. Our pathway enrichment results also suggest that the functional network of LRRC19 participates in peroxisome (Figure 7c) and fatty acid degradation, which had the highest NES scores (Figure 7d). The main function of peroxisome is to catalyze the β-oxidation of fatty acids, decompose very-long-chain fatty acids into short-chain fatty acids, and it is mainly present in the liver and kidneys. Interestingly, Glutathione peroxidase 3 (GPX3) is a member of the glutathione peroxidase family [71], involved in fatty acid hydroperoxide, cutting down the accumulation of hydrogen peroxide in the human body [72,73,74,75]. Abnormal inactivation or downregulation of GPX3 may lead to tumorigenesis caused by o excessive reactive oxygen species (ROS) including hydrogen peroxide, which may induce tumorigenesis [76,77,78]. Therefore, our analysis suggests that GPX3 is an important downstream gene of LRRC19, and that LRRC19 acts through this factor to regulate the lipid metabolism of KIRC. Further studies should test this hypothesis.

## 5. Conclusions

Through a comprehensive data mining for selenoproteins based on TCGA KIRC dataset, we screened out DIO1 and GPX3 as key genes associated with KIRC. A series of association analysis was performed and LRRC19 was found as an independent prognostic factor in KIRC. Pathway enrichment analysis indicated that LRRC19 could serve as a promising novel biomarker for prognosis and adjuvant treatment of selenium in KIRC. Our work provides significant insights into studies of selenium adjuvant therapy and renal clear cell carcinoma.

## Figures and Tables

**Figure A1:**
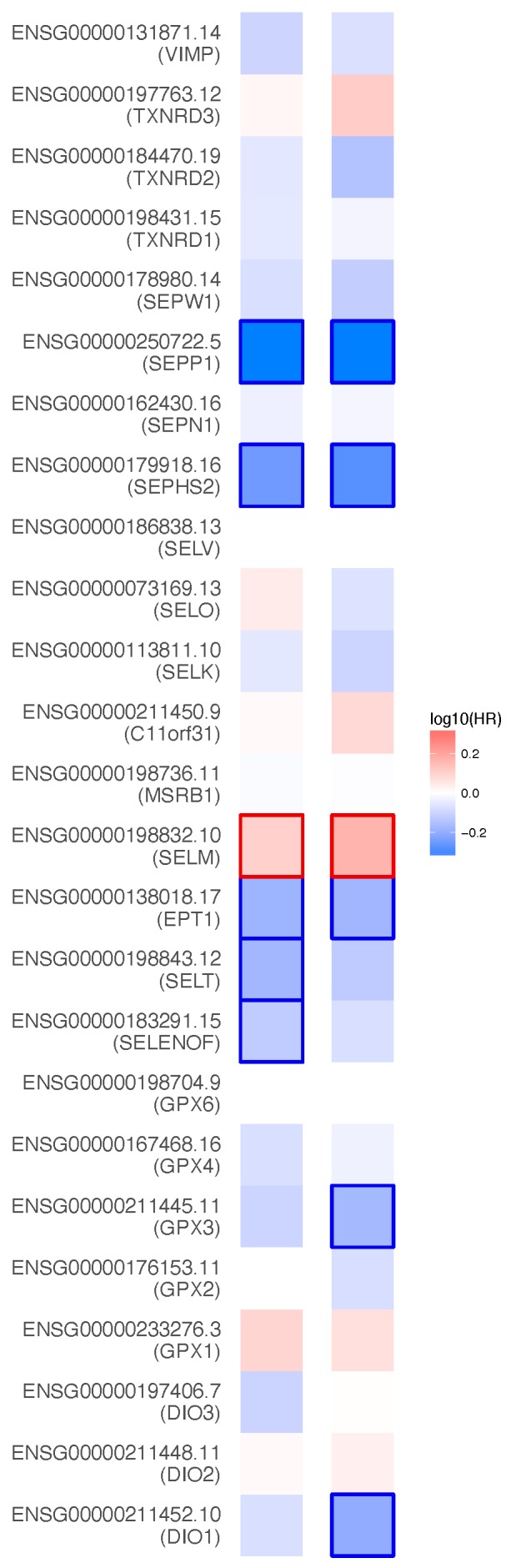
Heat map of $\log_{10}\left(HR\right)$ illustrating KIRC-selenoproteins pairs with altered prognosis (GEPIA2). We selected median as the suitable expression threshold for splitting the high-expression and low-expression cohorts. The hazards ratio (HR) was calculated based on Cox PH Model, and the HR value was scaled in decibel (dB). The red and blue color presented poor and good prognosis, respectively, and the intensity of color indicated the value of HR. The bounding box around the tiles represented whether there was statistical significance of the survival probability between the high and low expression groups in each cancer types (*p* < 0.05). From the OS and DFS results, SEPP1, SEPN1, SEPHS2, SELM, EPT1, SELT, SELENOF, GPX3, and DIO1 were screened out for their prognostic value in renal clear cell carcinoma. Overall survival (OS), Disease-free survival (DFS).

**Figure A2:**
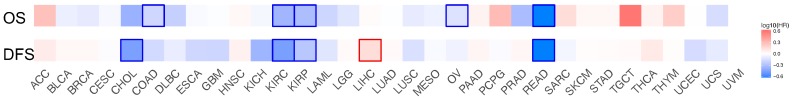
Survival analysis of LRRC19 in 33 cancer types of TCGA. Heat map of $\log_{10}\left(HR\right)$ was based on gene expression and survival time performed by GEPIA2. We selected 50% as the suitable expression threshold for splitting the high-expression and low-expression cohorts. The hazards ratio (HR) was calculated based on Cox PH Model, and the HR value was scaled in decibel (dB). The red and blue color presented poor and good prognosis, respectively, and the intensity of color indicated the value of HR. The bounding box around the tiles represented the statistical significance of LRRC19 in the cancer of the Axis Units (*p* < 0.05). From the results, LRRC19 was showed for its prognostic value in Cholangiocarcinoma (CHOL), Colon Adenocarcinoma (COAD), Kidney Renal Clear Cell Carcinoma (KIRC), Kidney Renal Papillary Cell Carcinoma (KIRP), Liver Hepatocellular Carcinoma (LIHC), Lung Adenocarcinoma (LUAD), Ovarian Serous Cystadenocarcinoma (OV), Rectum Adenocarcinoma (READ). Hazard ratios (HR), Overall survival (OS), Disease-free survival (DFS).

**Figure A3:**
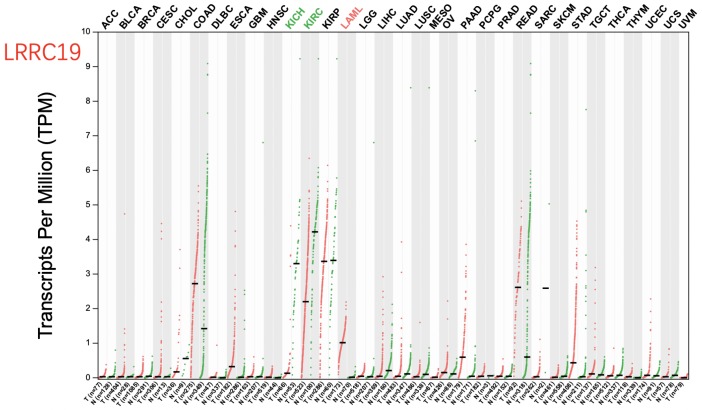
Dot plot profiling LRRC19 expression across 33 cancer types of TCGA and paired normal samples, with each dot representing a distinct tumor or normal sample. "TCGA normal + GTEx normal" were selected for matched normal data in plotting. The custom fold-change threshold was set as $|\log_{2}FC|>1$ and adjust.p-value < 0.01, and the expression data were $\log_{2}\left(TPM+1\right)$ pre-transformed. Each dot represents a distinct tumor (pink) or normal sample (green). The abscissa represents the sample from tumor tissue (T) or normal tissue (N), and “n” equals the sample size. The ordinate represents the amount of transcript expression in the sample. Above the table were the TCGA abbreviations of the cancer name. The cancer name coding in green indicated that the target gene was relatively lower expressed in the tumor tissue, and the red indicates higher expression. We selected LIMMA as the differential method. |$\log_{2}FC$| > 1 and FDR < 0.01 were considered as differentially expressed.

**Figure A4:**
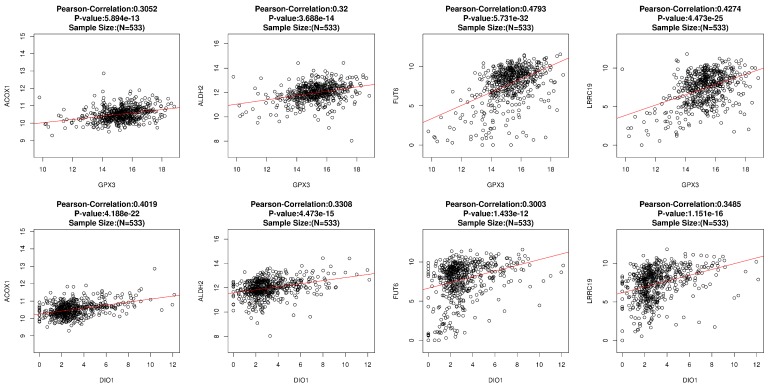
The correlation analysis of GPX3 and DIO1 was performed by the LinkFinder module of LinkedOmics. ACOX1, ALDH2, FUT6, and LRRC19 were the intersection genes generated from the GPX3 association results, DIO1 association results, and top 500 survival genes of KIRC. We used the Pearson Correlation test as the statistical method. Scatter plots showing associations with the four individual genes in detail. *p* < 0.01 and Pearson correlation coefficient *R* > 0.3 were considered as the two genes were statistically correlated in KIRC.

## Supplementary Materials

The following are available online at https://www.mdpi.com/2073-4425/11/4/440/s1, Table S1: Association results of GPX3 and DIO1 ( *LinkFinder*), Table S2: Most survival genes in KIRC (GEPIA2), Table S3: Univariate and multivariate Cox analyses.

## Appendix A. Prognostic Value of 25 Selenoproteins in KIRC

The survival maps in Figure A1 show the Hazard ratios (HR) of the 25 selenoproteins in KIRC.

## Appendix B. Prognostic Value of LRRC19 in Pan-Cancer

The heat maps in Figure A2 show the prognostic value of LRRC19 in pan-cancer.

The dot plot in Figure A3 shows the gene expression of LRRC19 in pan-cancer.

## Appendix C. Correlation Analysis of Intersection Genes

The scatter plots in Figure A4 show the correlation results for individual genes based on the KIRC dataset (LinkedOmics).
